# Isolation, Characterization, and Molecular Modeling of a Rheumatoid Factor from a Hepatitis C Virus Infected Patient with Sjögren's Syndrome

**DOI:** 10.1155/2013/516516

**Published:** 2013-12-30

**Authors:** Yu-Ching Lee, Keng-Chang Tsai, Sy-Jye Leu, Tuan-Jen Wang, Chia-Yu Liu, Yi-Yuan Yang

**Affiliations:** ^1^The Institute for Cancer Biology and Drug Discovery, College of Medical Science and Technology, Taipei Medical University, Taipei 110, Taiwan; ^2^Antibody and Hybridoma Core Facility, Taipei Medical University, Taipei 110, Taiwan; ^3^National Research Institute of Chinese Medicine, Taipei 110, Taiwan; ^4^Graduate Institute of Medical Sciences, College of Medicine, Taipei Medical University, Taipei 110, Taiwan; ^5^Department of Microbiology and Immunology, School of Medicine, College of Medicine, Taipei Medical University, Taipei 110, Taiwan; ^6^Department of Laboratory Medicine, Mackay Memorial Hospital, Taipei 104, Taiwan; ^7^Department of Clinical Pathology, Cheng Hsin Rehabilitation Medical Center, Taipei 112, Taiwan; ^8^Jen-Teh Junior College of Medicine, Nursing and Management, Miaoli County 356, Taiwan; ^9^School of Medical Laboratory Sciences and Biotechnology, College of Medical Science and Technology, Taipei Medical University, No. 250 Wu-Hsing Street, Taipei 110, Taiwan; ^10^Department of Laboratory Medicine, Wan Fang Hospital, Taipei Medical University, Taipei 110, Taiwan

## Abstract

We have previously isolated several IgG rheumatoid factors (RFs) from patients with both rheumatoid arthritis and idiopathic thrombocytopenia purpura using phage display system. To study IgG RFs in patients with other autoimmune diseases, phage display antibody libraries from a hepatitis C virus infected patient with Sjögren's syndrome were constructed. After panning, a specific clone RFL11 was isolated for characterization in advance. The binding activity and specificity of RFL11 to IgG Fc fragment were comparable to those of RFs previously isolated. The analysis with existed RF-Fc complex structures indicated the homology model of RFL11 is similar to IgM RF61 complex with high binding affinity of about 6 × 10^−8^ M. This effect resulted from longer complementarity-determining region (CDR) combining key somatic mutations. In the RFL11-Fc interfaces, the CDR-H3 loop forms a finger-like structure extending into the bottom of Fc pocket and resulting in strong ion and cation-pi interactions. Moreover, a process of antigen-driven maturation was proven by somatically mutated VH residues on H2 and H3 CDR loops in the interfaces. Taken together, these results suggested that high affinity IgG RFs can be generated in patients with Sjögren's syndrome and may play an important role in the pathogenesis of this autoimmune disease.

## 1. Introduction

Sjögren's syndrome (SS) is an autoimmune disorder that mainly affects the exocrine glands and usually presents as persistent dryness of the mouth and eyes due to functional impairment of the salivary and lachrymal glands [1]. SS occurs in a primary form not associated with other diseases and in a secondary form that complicates other rheumatic conditions, with the most common being rheumatoid arthritis. Positive RF was found in 96% of the patients with main extraglandular SS [2]. On the other hand, circulating monoclonal immunoglobulins (IgM kappa or IgG lambda) were detected in a significant higher frequency (43%) of SS-HCV patients as compared with the primary SS patients [3]. Hepatitis C virus (HCV) has been demonstrated to be one of the most likely candidates as a potential pathogenic agent causing SS in a subset of patients [2, 4, 5]. Many rheumatologic manifestations associated with chronic HCV infection include arthralgia, myalgia, arthritis, vasculitis, and sicca syndrome [6]. Clinical studies suggest the possibility of a close relationship among SS, HCV, and B-cell lymphoproliferative disorders [2, 4]. This triple association suggests an important role of associated autoimmune and/or chronic viral diseases in the pathogenesis of B-cell lymphoproliferative disorders and reinforces the hypothesis of a link among autoimmunity, infection, and cancer [4].

Rheumatoid factors (RFs) are antibodies directed against the Fc part of autologous IgG and are the most characteristic marker in rheumatoid arthritis (RA), a chronic joint inflammation with unknown etiopathogenesis [7, 8]. Complex formation between RF and IgG may lead to activation of complement and other inflammatory mediators directly [9]. Physiological RF mainly belongs to IgM isotype. It serves a beneficial role in host defense which facilitates the clearance of antigen by enhancing complement activation and phagocytosis. Oppositely, pathological RF is associated with RA and other systemic autoimmune diseases [10, 11]. Monospecific IgG RFs are implicated in causing inflammation and tissue damage in the rheumatoid synovium [1, 7]. Corper et al. were the first group to visualize RF binding by crystal structure directly showing an epitope spanning the junction of the C**γ**2 and C**γ**3 domains on Fc fragment [12]. In another study, Duquerroy et al. determined a different epitope recognition site using their IgM RF [13]. Their definite C**γ**3-C**γ**3 interface, including the C-terminal region of both heavy chains, is distant from the previously identified C**γ**2-C**γ**3 interface [12]. Both complexes were generated from IgM RF origin and indicated the involvement of somatic mutations in the interaction interface.

Human monoclonal antibodies were in great demand for the therapeutic and diagnostic purposes. The combinatorial methodologies have been improved rapidly and dramatically [14, 15]. Particularly, the expression of combinatorial antibody Fab fragments on phage surfaces allows for rapid enrichment of the desired Fab fragments against target antigens. This technique has been used to study pathogenic autoantibodies in various human autoimmune disorders [16–18]. To study IgG RFs in Sjögren's syndrome patients with HCV infection, we used the combinatorial antibody technique and characterized isolated RF Fab molecules. The results allow us to further define the pathogenic role of RFs played in autoimmune diseases complicated with viral infection. In addition, from the insight on the genetic level, clues are expected to reveal the association of the cause of autoimmune disorders.

In order to comprehensively explain the interactions between an antibody and its target antigen, molecular modeling has been employed as a powerful and reliable approach for rational model prediction of an antibody-antigen complex. By analyzing and comparing the X-ray crystal structures of antibody-antigen complexes retrieved from the Protein Data Bank (PDB), many researches have demonstrated that sufficient information is available for analysis to indicate critical residues of great influence on the binding sites from antibody-antigen recognition interfaces [19]. In this study, we demonstrated that the isolated IgG RF has a better binding affinity than the existing complex generated by the IgM RF molecule. We managed to construct antibody models via homology modeling and used structural alignment and loop minimization to illustrate the possible role of somatic mutation induced in the CDR areas during the interactions. The logical and reasonable predicted results provide a probable explanation and further insights into the diversity and origin of these IgG RFs.

## 2. Material and Methods

### 2.1. Antibody Library

Two antibody libraries were constructed to express antibody binding fragment (Fab) from patients with Sjögren's syndrome associated with hepatitis C infection. Library sizes for **κ** and **λ** light chain were 1.3 × 10^7^ and 2.1 × 10^6^, respectively. Equal amount of phage particles was taken from two libraries and mixed evenly for subsequent panning cycles.

### 2.2. Panning and Identification of Human Fc Binders

The antigen-binding clones in the prepared library were enriched by panning on antigen-coated surface of ELISA plates (Costar) as reported previously [20, 21]. Briefly, human Fc fragment protein (Sigma) was coated as target protein with 0.5 ug/well at 4°C overnight. After blocking with 5% skim milk, 10^11^ pfu of recombinant phages were added to each well and incubated at 37°C for 1 hr. Unbound phages were removed and the wells were washed vigorously with Tris-buffered saline containing 0.05% Tween-20 (TBST) for ten times. Next, bound phages were eluted with 0.1 M HCl/glycine (pH 2.2) and neutralized with 2 M Tris-base. Eluted phages were used to infect *E. coli* XL1-blue strain growing in log phase. Phagemid particles were rescued from infected *E. coli* cells with 10^11^ pfu of VCS-M13 helper phage (Stratagene). After culture amplification, 4% PEG-8000 and 3% NaCl were used to precipitate recombinant phage particles. Finally, the phages were resuspended in PBS and used for the next round of panning. Panning processing against human Fc fragment was repeated four times. Thereafter, total phagemid DNA was prepared and digested with *Nhe* I and *Spe* I (NEB Biolab) to remove the phage protein III gene. The digested DNA with compatible cohesive ends was self-ligated and electroporated into *E. coli* XL1-blue cells. Individual clone was grown overnight in the presence of 0.5 mM isopropyl b-D-thiogalactopyranoside (IPTG) for Fab protein induction. The supernatants containing expressed Fab molecules were harvested for ELISA and Western blotting assay.

### 2.3. Enzyme-Linked Immunosorbent Assay (ELISA)

Briefly, microtiter plates were coated with 0.5 ug/well with human Fc fragment protein at 4°C overnight. The wells were blocked with 5% skim milk for 1 hr at room temperature. The expressed Fab molecules prepared as described above were distributed to wells in duplicate and incubated for 1 hr at room temperature. After washing with PBS containing 0.05% Tween-20 (PBST) six times, the wells were reacted with horseradish-peroxidase- (HRP-) conjugated goat anti-human **λ** or **κ** light chain antibodies (Jackson ImmunoResearch Laboratories) for 1 hr at room temperature. The wells were then washed with PBST six times and 3,5,5-Tetramethubezidine dihydrochloride (TMB) substrate was added for color development. Reaction was stopped by addition of 1 N HCl and absorbance was taken at 450 nm.

For characterization of binding specificity, the selected Fc-binding Fab was studied their reactivities with type VI collagen (Sigma), lipopolysaccharide (LPS; Sigma), keyhole limpet haemocyanin (KLH; Sigma), thyroglobulin (Sigma), and calf thymus single-stranded DNA (ssDNA; Sigma) simultaneously. The ELISA analysis was carried out as described above. The amount of these antigens used for coating was 0.5 ug/well.

### 2.4. Western Blotting

To examine the Fab expression, the IPTG-induced bacterial lysates were subjected to sodium dodecyl sulfate polyacrylamide gel for electrophoresis (SDS-PAGE) and transferred onto nitrocellulose membrane (GE Biosciences, UK), followed by blocking with 5% skim milk in PBST for 1 hr at room temperature with gently shaking. After washing, HRP-conjugated goat anti-human **λ** or **κ** light chain antibodies were added and incubated for one additional hour at room temperature with gently shaking. After washing with PBST, the membranes were developed with diaminobenzidine (DAB) substrate until desired intensity was reached.

To examine their binding ability, the cellular lysates containing recombinant Fab molecules or sera from HCV-infected patients with Sjögren's Syndrome were incubated with human Fc fragment fixed on Western blots. After human Fc fragments were transferred onto nitrocellulose membranes, the experiments were performed as described above.

### 2.5. Competitive Inhibition Assay of Fc-Binding Fabs

Competitive inhibition assays were carried out to determine the binding affinities of Fc-binding Fab molecules. In brief, microtitre plates were coated with human Fc fragment (0.5 **μ**g/well) and blocked with 5% skim milk. After one hour incubation at room temperature, plates were washed with PBST twice. Each purified Fab was incubated first with various concentrations of soluble Fc fragment diluted in PBS (ranging from 2.5 to 80 **μ**g/mL) at room temperature for 1 hr. Then, the mixtures were added to the wells to react with coated human Fc fragment. After incubating at room temperature for 1 hr, plates were washed with PBST six times. Thereafter, HRP-conjugated goat anti-human **λ** or **κ** light chain antibodies (Jackson ImmunoResearch Laboratories) were incubated at room temperature for 1 hr to detect bound Fab protein. After washing with PBST six times, TMB substrate (Sigma) was added to each well for development. Reaction was stopped with 1 N HCl and signal intensity was measured at OD 450 nm.

### 2.6. DNA Sequence Analysis

Light and heavy chain inserts of Fc-binding clones were sequenced and analyzed. Primers complementary to constant region of heavy chain (SeqGb: 5′-GTC GTT GAC CAG GCA GCC CAG-3′ (21 mers)) and lambda light chain (SeqLb: 5′-GAA GTC ACT TAT GAG ACA CAC-3′ (21 mers)) were used. The IMGT/V-QUEST international immunogenetics information system (http://www.imgt.org/) was used to compile and analyze sequence data in parallel with germline gene.

### 2.7. Molecular Modeling

RFL11, an anti-human IgG Fc Fab, was found to possess the highest affinity among several clones screened in this study and modeled as an scFv scaffold using the homology modeling program. To obtain a reliable light chain model for RFL11, sequence alignment of the **λ** light chains of RFL11 and RF61 antibodies (PDB id: 2J6E) [13] was used, showing 97.6% or 92.4% of homology, exclusive or inclusive of CDRs, respectively. Similarly, to build the heavy chain model of RFL11, the heavy chain sequence showed a high similarity when compared with another antibody (PDB id: 3H0T) [22], exclusive (98.8%) or inclusive (91.7%) CDRs.

Homology model of RFL11 was generated by MODELER v.9.4 program in Discovery Studio v.3.5 (Accelrys Software Inc., San Diego, CA, USA) using the crystallographic structures of the **λ** light chain of RF61 (PDB id: 2J6E) and the heavy chain of the antibody (PDB id: 3H0T) as structural templates. Both structures have good resolution and belong to *Homo sapiens*. Thus, the interface of the template structure between L chain and H chain of RF61 was employed to build the light chain–heavy chain interface of RFL11 scFv model.

Owing to the **λ** light chains of RFL11 and RF61 antibodies with high homology CDRs, RFL11 and RF61 may bind to the similar epitope of IgG Fc (see Table 1). The complex structural model of RFL11 interacting with IgG Fc antigen was built by aligning RFL11 and the complex structure of RF61-IgG Fc to obtain an RFL11-IgG Fc complex instead of using docking methods [23] to avoid uncertain orientation of the interaction between these two proteins. The CDRs of RFL11 interacting with IgG Fc antigen were energy minimized using Dreiding-like force field from Discovery Studio v.3.5 for a few steps to remove any inappropriate contacts and obtain a reliable complex structure.

## 3. Results

### 3.1. Panning and Characterization of the Isolated Clones

Two Fab-displaying phage libraries were constructed from a hepatitis C virus infected patient with Sjögren's syndrome. The numbers of heavy chain combined with either **λ** or **κ** light chain were 2.1 × 10^6^ and 1.3 × 10^7^, respectively. To evaluate the ratio of clones lacking correct inserts in the libraries after amplification, fifty clones of each library were selected for restriction analysis to confirm the presence of a 750 bp fragment containing both heavy and light chain genes. The results revealed that over 93% (14/15) of the clones selected from two libraries had correct gene inserts (data not shown).

**Figure 1:**
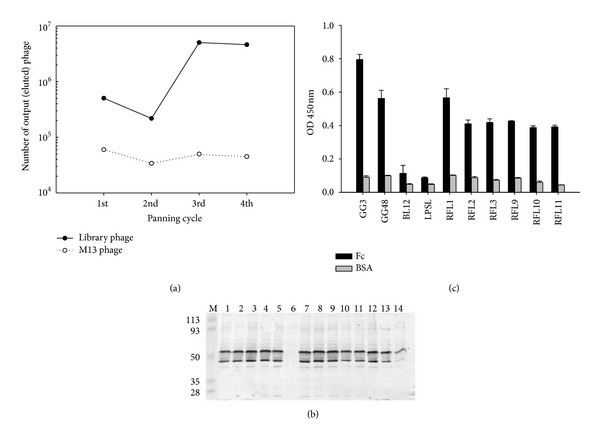
(a) Eluted phage titers after each round of panning. (b) Western blotting analysis of IPTG-induced Fab antibody expression under nonreducing condition. The protein patterns were visualized using goat anti-human **λ** light chain antibodies. (c) Randomly selected clones from 4th panned library were examined for their binding to human Fc fragment by ELISA. GG3 and GG48 denoted previously isolated clones expressing recombinant Fab molecules with Fc-binding activity. BL12 and LPSL are negative controls of irrelevant Fab molecules expressed in *E. coli*. Bound Fab was detected using goat anti-human **λ** light chain antibodies.

The Fc-binding phages were enriched by four rounds of panning. The input phage number of 10^11^ was applied for each panning processing. It was generally seen that the eluted phage titer of final round was about one order higher than that of first panning (Figure 1(a)). After each panning, 15 clones were randomly selected to check the presence of DNA inserts with correct length. The results showed that more than 90% of the clones contain a fragment of 750 bp in plasmid DNA after second around panning (data not shown), indicating that the panning eliminated most of the phage clones not displaying Fab fragments on their surfaces, which accounted for less than 10% of phage clones in the original antibody library we constructed.

Thereafter, the gene III fragment was removed from phagemid DNA purified from *E. coli* culture after 4th panning. The expression of IPTG-induced Fab antibodies under the nonreducing condition was clearly detected and shown in Figure 1(b). With the exception of one clone with incomplete nucleotide sequence, the immunoglobulin isotyping anaysis indicated that 13 of 14 RF molecules contained **λ** light chain. We did not find any antibody with **κ** light chain.

### 3.2. Binding Specificity and Affinity Determination against Human Fc

**Figure 2:**
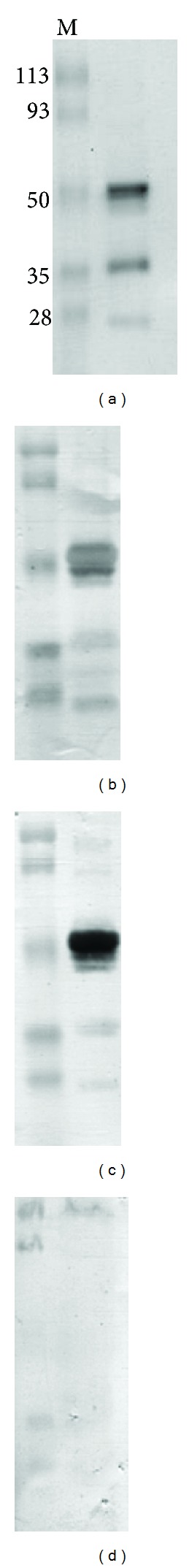
The Fc-binding reactivity of RFL11 clone using Western blot analysis. Panel (a) showed human Fc fragment on a Coomassie blue-stained polyacrylamide gel. After transferred onto the nitrocellulose membrane, the Fc fragments were detected by serum of HCV-infected patient with Sjögren's syndrome (b) and RFL11 (c) but not an irreverent Fab molecule in cellular lysate (d).

**Figure 3:**
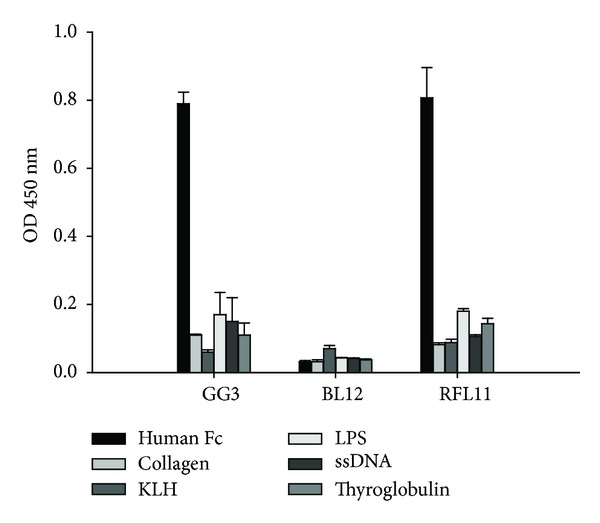
The binding specificities of the RFL11-Fc binder. Fab molecules were analyzed comparatively against Fc fragment and a panel of five unrelated antigens, including collagen, KLH, LPS, ssDNA, and thyroglobulin. GG3 and BL12 denoted positive and negative Fab controls, respectively.

All the clones containing **λ** light chain of Fab were further tested for binding activity and specificity. GG3 and GG48 clones with high binding specificity to Fc [24] and LPSL and BL12 clones with anti-LPS and anti-red-blood surface Ag were applied as positive and negative controls, respectively. These selected Fab fragments showed significant binding activity to Fc but not to BSA (Figure 1(c)). Moreover, their recognition of human Fc fragment immobilized on Western blot was tested. The results showed both serum of HCV-infected patient with SS disease (Figure 2(b)) and the recombinant Fab molecule as represented by RFL11 (Figure 2(c)) could clearly recognize Fc protein. No Fc-binding activity was observed when an irreverent Fab molecule was applied (Figure 2(d)). In order to test the binding specificity, unrelated antigens including collagen, KLH, LPS, calf thymus ssDNA, and thyroglobulin were used in ELISA test. As shown in Figure 3, the representative RFL11 Fab antibody as well as the positive GG3 clone showed specific binding activity to Fc fragment but minimal binding to the panel of unrelated antigens.

**Figure 4:**
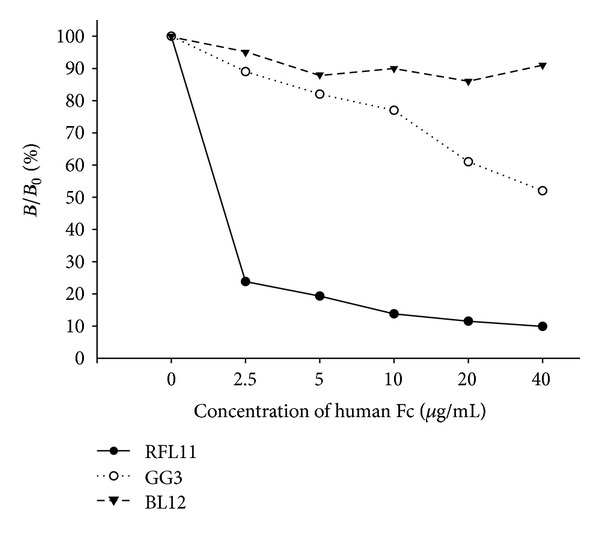
Competitive inhibition assay of RFL11 Fab antibody against the Fc fragment. The amount of bound Fab in the presence of free Fc inhibitor was measured and expressed as a percentage of the binding of Fab in the absence of an inhibitor. B and B0 denoted the amount of bound Fab in the presence and absence of the inhibitor, respectively.

The binding specificity and affinity of RFL11 was further determined by competitive inhibition assay. As demonstrated in Figure 4, 89% inhibitory effect was found on the binding reactivity of RFL11 Fab against coated human Fc fragment in the presence of a concentration of 40 ug/mL of free Fc fragment. When performed in parallel, close to 50% of inhibitory effects were seen using positive GG3 Fab antibody and none were detected using negative BL12 Fab molecule. The *K*_*d*_ value for RFL11 antibody was 6 × 10^−8^ M calculated using the Klotz plot method [25].

### 3.3. Sequence Analysis of Isolated Fc-Binding Clone

**Figure 5:**
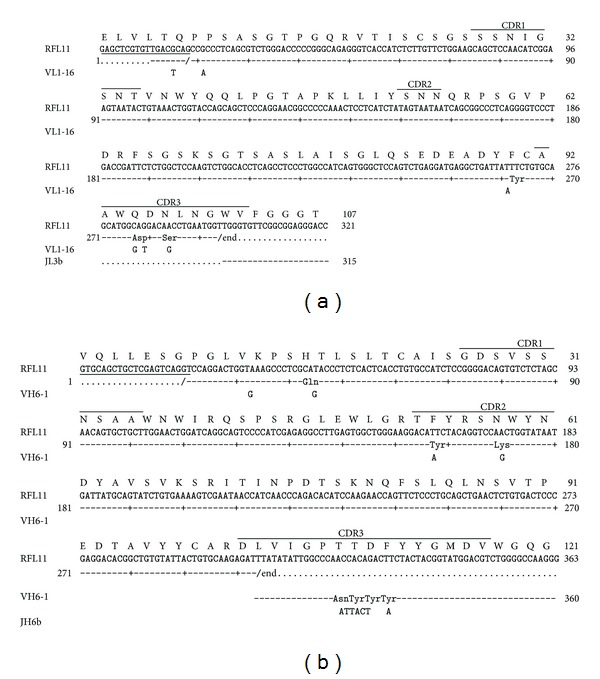
Sequence analysis of variable regions of light chain (VL) in the panel (a) and heavy chain (VH) in the panel (b) of RFL11 Fab antibody. The sequences of putative germline counterpart were included for comparison. Sequence gaps were introduced to maximize alignment as indicated by blank spaces. The boundaries of framework region (FR) and complementarity-determining region (CDR) were indicated above each germline gene sequences.

The nucleotide sequences of VH and VL region of Fc-binding clones were determined. When compared to potential germline, the light chain and heavy chains of RFL11 were determined and shown the highest homology to VL1-16 and VH6-1 (Table 2). Three nucleotide differences were identified in CDR3 region of the light chain gene. Two changes in nucleotide positions 277 and 279 (GAT to CAG) resulted in amino acid residue that changed from Aspartate (D) to Glutamine (Q). The third change in nucleotide 284 (AGC to AAC) led to amino acid residue that changed from Serine (S) to Asparagine (N) (Figure 5(a)). Similarly, two nucleotide changes in 158 and 171 of CDR2 of the heavy chain of RFL11 mutated Tyrosine (Y) to Phenylalanine (F) and Lysine (K) to Asparagine (N), respectively. As compared to JH6b germline gene, four continual residues were somatically mutated making NYYY to TTDF in CDR3 region (Figure 5(b)).

**Figure 6:**
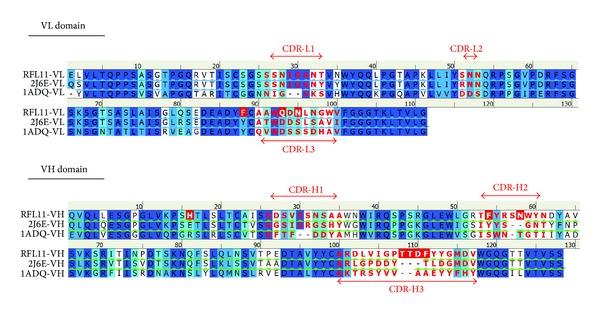
Multiple sequence alignment of variable domains of the light and heavy chains of RFL11 with 2J6E and 1ADQ antibodies. The background of the residues is colored according to sequence similarity. Deep blue color shows conserved residues in all sequences. The color scheme is from deep blue and light blue to cyan corresponding to identify highly and low conserved residues, respectively. The residue backgrounds colored white are not similar. Residues of somatic mutation of RFL11 have a red background. Residues of the CDRs are indicated by bold letters in red.

Compared to sequences in the CDR domains of RF61 RF molecule, light chain shows the most similarity except CDR3. RFL11 has replacement of Aspartate (D) and Serine (S) with Glutamine (Q) and Asparagine (N). For heavy chain, significant dissimilarity on CDR was shown on CDR2 and CDR3 with additional residues. Besides, the framework of RFL11 to 2J6E was high similarity (Figure 6).

### 3.4. The Interaction of the RFL11 scFv Antibody with Human IgG Fc Antigen

To investigate the interaction of RFL11 with IgG Fc, the six CDRs of RFL11, all of which make contact except CDR-L2 loop and the H2 and H3 loops of RFL11 that dominate the interaction with Fc, are shown in Figure 8. From the interaction between CDR-L2 loop and IgG Fc, we found that Phe85 side chain and Tyr49 side chain on the H2 loop correspondingly act on Asp356 and Lys439 on the IgG Fc, respectively, and the interactions involved are anion-pi interaction and cation-pi interaction, respectively. In addition to the two interactions, some H-bondings are also involved, as exemplified below. The side chain of Arg50 acts on the side chains of Gln438 and Tyr436 via two H-bondings; the side chains of Ser51 and Asn52 interact with the main chain of Gln438 and produce H-bondings; moreover, an H-bonding is also produced between the side chain of Asn52 and the side chain of Gln438.

The CDR-H3 loop forms a finger-like structure extending into the bottom of a deep pocket of the IgG Fc, which is composed of Arg355, Ser442, Pro352(L), and Pro352(H). Four different interactions are generated by the H3 loop and Fc, namely, ion interaction, cation-pi interactions, H-bonding, and hydrophobic interactions. The IgG Fc-binding surface contains a main charged residue Arg355 which is involved in the ion interaction to Asp104 and the cation-pi interactions to Tyr106 and Tyr107 on the CDR-H3 loop (Figure 8). Moreover, the side chain of Phe105 on the CDR-H3 loop interacts with Pro352 in the H/L chain of C*γ*3 domains via two hydrophobic contacts. The Thr102 side chain and Thr103 side chain on the CDR-H3 loop, respectively, interact with Ser444 and Ser442 on the IgG Fc, both resulting in the formation of H-bonding.

## 4. Discussion

In this study, we described the isolation and characterization of RFL11 Fab directed against human IgG Fc from a patient with Sjögren's syndrome and HCV infection. Being different from physiological RF arising from normal antibody responses to aid exogenous antigen clearance, these RFs may be involved in the pathogenesis of the patients with SS/HCV infection. The isolated IgG RF was proven to bind specifically to human IgG Fc fragment. The binding affinity was 6 × 10^−8^ M, as determined by competitive inhibition assays (Figure 4). Moreover, subsequent result of an additional serum sample from a patient with Sjögren's syndrome revealed the presence of IgG RFs (Figure 2). Although phage-derived Fabs may represent the artefacts of recombination of H and L chains during library construction, the characteristic of isolated RFL11 could still be a hint to verify the existence of high affinity RF in patients with Sjögren's syndrome. Our data suggest that IgG type RFs may occur in some patients with Sjögren's syndrome and that such IgG type RFs may have binding affinities higher than those of IgM or IgG RFs isolated from other autoimmune diseases [12, 13, 26].

To further study and characterize these pathogenic RFs, it is important to comprehend the interaction between RF and Fc as well as to realize the influence of somatic mutations on antibody genes. After a comparison of the heavy chain and light chain variable gene sequence with germline counterpart, RFL11 was identified by ten probable somatic mutations, two in frameworks (Tyr88Phe (VL) and Gln16His (VH)) and eight in CDRs (Asp93Gln and Ser95Asn (L3); Tyr54Phe and Lys58Asn (H2); Asn108Thr, Tyr109Thr, Tyr110Asp, and Tyr111Phe (H3)) (Figure 6). Unlike the antibody gene variations isolated in our previous works, where a substantial amount of somatic mutations occurs in CDRs or even in the framework regions in order to fight against exogenous pathogens, these pathogenic RFs caused by immune system disorders, however, possess a sequence similar to the endogenous germline sequence on the genetic level, except that critical mutations occur on the CDRs to provide a binding-specific ability to the IgG Fc domain. This implies that the generation of RF autoimmune antibodies is attained by critical changes which convert physiological RFs into pathogenic RFs.

In general, output phage value after enrichment implies the success of the panning process if greater than 50-fold [27]. At lower enrichment levels, nonspecific and low affinity binders could cause outgrowth in the output phage after amplification. Moreover, in the panning process, although eluted phage titer with mild increasing only raised one order of magnitude after final round of panning, we still enriched and isolated specific phage binders from the original library successfully (Figure 1(a)). It is interesting to show that the output phage numbers in our panning process had instant increment after the third cycle, although it dropped slightly in the second round (Figure 1(a)). This result agrees with other studies [28, 29]. After sequencing of isolated clone following the final panning, almost all clones were identified to be similar. It is another verification to convince an efficient panning process resulting in high affinity clone to become dominant in the final library pool.

From the results of competitive inhibition assay, a high binding affinity of RFL11 Fab having anti-human IgG Fc capability was confirmed. RFL11 shares high similarity with the L chain amino acid sequence of RF61 autoantibody, which has been reported, but is different from another autoantibody (PDB id: 1ADQ, lower binding affinity *K*_*d*_ ~ 6 × 10^−5^ M) already published [12]. In addition, CDRs H2 and H3 on the H chain of RFL11 have two and three more amino acids than those of RF61, respectively, but the amino acid sequences on the frameworks are highly similar. Despite the fact that RFL11 and RF61 come from different immunoglobulin isotypes (IgG and IgM types, resp.), their antigens are both IgG Fc; accordingly, it can be reasonably inferred that the epitopes recognized by RFL11 and RF61 have a high similarity, and the docking simulation of RFL11 antibody and IgG Fc antigen by using zDOCK program [23] also revealed an epitope highly similar to that corresponding to RF61. In order to establish a reliable structure model of RFL11 antibody-Fc complex, we used structural alignment and CDR loop minimization to replace docking. From this approach, the dominating interactions of H2 and H3 loops between RFL11 and IgG Fc can be more clearly observed to further investigate why RFL11 demonstrates a superior binding affinity to RF61, as indicated from the experiments.

Table 1 illustrates the epitope residues on IgG Fc. Gln438 and Lys439 on Fc individually form H-bonding and cation-pi interactions with the CDR-H2 loop, so we can infer that Gln438 and Lys439 are key residues for both RFL11 and RF61 on the IgG Fc epitope. However, from the paratope of the CDR-H2 loop, Asn52 and Phe48 on the RFL11 CDR-H2 loop, generated by somatic mutation, respectively, form H-bonding and anion-pi interactions with respect to Gln438 and Asp356, indicating the importance of somatic mutation to the CDR-H2 loop. Compared with the RF61 CDR-H2 loop, the two additional amino acids Ser51 and Asn52 on the RFL11 CDR-H2 loop both interact with Gln438 on Fc with a strong H-bonding, showing that the RFL11 CDR-H2 loop has a binding affinity stronger than the RF61 CDR-H2 loop to IgG Fc.

**Figure 7:**
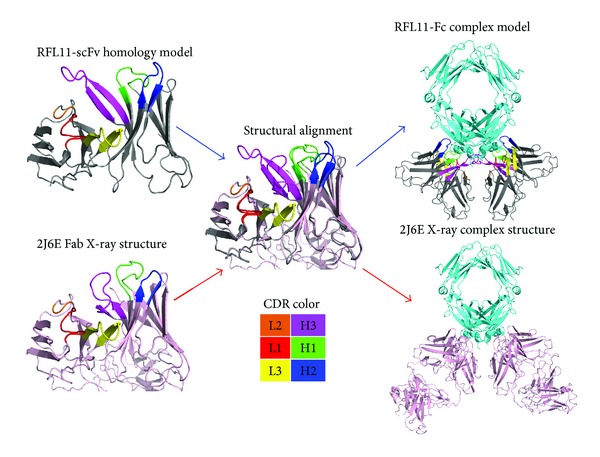
Comparison of RFL11 and RF61 (PDB id: 2J6E) complexes with human IgG Fc. The orientation of CDRs in both RFs was colored identically, except H2 and H3 loops. The affinity improvement by slight difference in the interactions was discussed particularly in advance.

**Figure 8:**
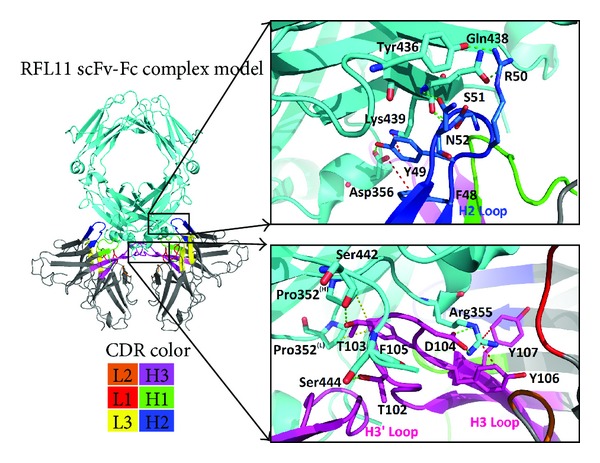
The interface structure of the RFL11-Fc was focused on the CDR-H2 (colored in blue) and CDR-H3 (colored in magenta) loops in this panel. Human IgG Fc antigen was colored in cyan. The colors of the six CDRs of RFL11 were the same as that in Figure 6. The format of residues of Fc antigen and H2/H3 loops is abbreviated as three words and residues codes for distinguishing between antigen and antibody, respectively. The green and red dotted lines in the above panel represent H-bonding and anion-cation interactions of RFL11 with Fc, respectively. In the below panel, the green, red, and yellow dotted lines show H-bonding, cation-pi, and hydrophobic interactions, respectively.

The RFL11 CDR-H3 loop has three more amino acids than the RF61 CDR-H3 loop, and these three amino acids belong to the four amino acids (Thr102, Thr103, Asp104, and Phe105) of RFL11 produced by somatic mutation. H-bonding and hydrophobic and ion interactions are formed between IgG Fc and the four amino acids, among which Asp104 forms a critical strong ion interaction with IgG Fc Arg355. Moreover, Tyr106 and Tyr107 also both contribute to the formation of cation-pi interaction with Arg355. As shown in Table 1, ion interaction is formed between RF61 and Arg355 of IgG Fc via Asp98 and Asp100c. Thus, Arg355 is a key residue to the IgG Fc epitope. Based on the comparison made according to Table 1, it is inferred that the RFL11 CDR-H3 loop has a binding affinity to IgG Fc similar to that of the RF61 CDR-H3 loop, but there might be some difference in the Fc epitope. Also, from the structure model, it is observed that the RFL11 CDR-H3 loop forms a finger-like structure extending into the bottom of a deep pocket of IgG Fc, which is different from the ellipse-like structure bound to the IgG Fc formed by RF61. Due to the finger-like structure, the CDR-H3 loops of RFL11 can interact with each other as a dimer and together form interactions with IgG Fc (See Figure 7), providing a binding affinity to the CDR-H3 loops of RFL11 higher than the RF61-H3 loops. From the binding structure of RFL11 and RF61 with Fc, although a novel epitope was not found, we confirmed that IgG type RF is different from IgM type RF and discovered a different CDR loop, particularly a substantial difference between H2 and H3 paratopes. Our study showed that RFL11 has a stronger binding affinity than RF61, and both the somatic mutation and computational structural biology investigation are in line with the experiments.

## 5. Conclusion

In this study, we illustrated that high specificity and high binding affinity RFs may possibly exist in SS patience. By analyzing the isolated RF sequences, we managed to explain the reasons why the isolated RFL11 may possess high affinity to IgG Fc from the structure and simulation perspectives. Although a direct relationship between the isolated RFs and autoimmune diseases has not been established, we have demonstrated how these meaningful RFs change the paratopes on CDR through somatic mutation to induce pathogenic high binding affinity and provided insightful information and guidance useful for future RF researches.

## Figures and Tables

**Table 1:** Comparison of contact residues in RF61 and RFL11 interaction with Fc.

	CDR residue	Fc contact	Fc chain	Types of contact
	(IMGT numbering)			
*RF*61		—		
H2	Tyr52 (s)	Lys439 (s)	A	Hydrophobic
		Ser440 (m)	A	H-bonding
	Tyr53 (s)	Gln438 (s)	A	H-bonding
		Ser440 (s)	A	H-bonding
	Ser54 (s)	Gln438 (m)	A	H-bonding
				
H3	Asp98 (s)	Arg355 (s)	B	Ion interaction
		Asp356 (s)	B	Anion-pi
	Tyr100 (s)	Thr350 (s)	A	H-bonding
		Lys439 (s)	A	Cation-pi
	Thr100a (s)	Ser440 (m)	A	H-bonding
		Arg355 (s)	B	H-bonding
	Asp100c (s)	Arg355 (s)	B	Ion interaction

*RF* *L*11				
H2	Phe48 (s)*	Asp356 (s)	A	Anion-pi
	Tyr49 (s)	Asp356 (s)	A	H-bonding
		Lys439 (s)	A	Cation-pi
	Arg50 (s)	Gln438 (s)	A	H-bonding
		Tyr436 (s)	A	H-bonding
	Ser51 (s)	Gln438 (m)	A	H-bonding
	Asn52 (s)*	Gln438 (m)	A	H-bonding
				
H3	Thr102 (s)*	Ser444 (s)	A	H-bonding
	Thr103 (s)*	Ser442 (s)	A	H-bonding
	Asp104 (s)*	Arg355 (s)	A	Ion interaction
	Phe105 (s)*	Pro352 (s)	A	Hydrophobic
		Pro352 (s)	B	Hydrophobic
	Tyr106 (s)	Arg355 (s)	A	Cation-pi
	Tyr107 (s)	Arg355 (s)	A	Cation-pi

Abbreviations: (s) and (m) denote the side chain and the main chain, respectively.

*Denotes the residue of somatic mutation.

**Table 2:** Putative germline analysis of Fc-binding clone RFL11.

Clone	VH germline		D	JH	VL germline		JL
	Putative	Homology (%)			Putative	Homology (%)	
RFL11	VH6-1	98.6	D4-23	JH6	V*λ*-16	97.9	JL1
